# Trauma-informed family carer education and practical skills training in dementia: a systematic scoping review protocol

**DOI:** 10.1136/bmjopen-2024-090202

**Published:** 2024-12-07

**Authors:** Eileen Harkess-Murphy, Debbie Tolson, Joshua Cheyne, Suzanne Heron, Anthony Butler, Yvonne Murray, Bryan Mitchell, Kath Limond

**Affiliations:** 1 Health and Life Sciences, University of the West of Scotland, Paisley, UK; 2 University of the West of Scotland, Paisley, Renfrewshire, UK

**Keywords:** Dementia, Dementia, Family, EDUCATION & TRAINING (see Medical Education & Training), Stress, Psychological

## Abstract

**Abstract:**

**Introduction:**

The incurable and progressive nature of dementia requires complex care, the majority of which is provided via informal caring by family members within the family home. Carers experience significant stress absorbing the challenging care needs of their family member and require education and training that can support and sustain family caring arrangements while considering the psychological distress that threatens caring breakdown. The aim of this scoping review was to map the evidence of trauma-informed principles within education and practical skills training in dementia family caring.

**Methods and analysis:**

A two-step approach to the selection of literature will be used. In step 1, the review will consider research on active intervention education and practical skills training to support family home-based informal care for individuals with a formal diagnosis of dementia. The review will exclude passive education and self-accessed information/training provision. Only literature in the context of ‘informal’ day-to-day family caring provided by a family member or friend that takes place in the family home or residence will be included. Education and practical skills training provision within specialist care environments will be excluded. In step 2, during the full-text screen, only research where either explicit or implicit use of trauma-informed approaches has been used will be included.

Preliminary searches of MEDLINE Ovid and Cumulative Index to Nursing and Allied Health Literature (CINAHL) were carried out between March and May 2023 to identify literature in this area. In line with the Johanna Briggs Institute scoping review guidance, we will conduct a search of published literature within MEDLINE Ovid, Embase Ovid, CINAHL EBSCO, Cochrane Data for Systematic Reviews and Cochrane Central Register for Controlled Trials in the Cochrane Library, PsycINFO Ovid and the British Library EThOS e-theses online. Publications in English with a date range of 1990 to current, with no restriction on geographical region will be considered. The search will be managed by Rayyan software and screened by multiple independent researchers. Results will be presented using narrative summaries and tables.

We collaborated with an experienced Academic Support Librarian to develop the MEDLINE Ovid search strategy (Appendix 1), which will be adapted for searching other databases.

**Ethics and dissemination:**

Ethical approval was not required for this review, as it involved the synthesis of publicly available secondary data. The findings will be disseminated through publication in peer-reviewed journals, as well as presentations at national and international conferences. Additionally, stakeholder events will engage carers, individuals with lived experience, and healthcare professionals.

**STRENGTHS AND LIMITATIONS OF THIS STUDY**

- The protocol has been registered with the Open Science Framework.
- An experienced academic support librarian, in collaboration with a multi-disciplinary team of nurse educators, dementia specialists, mental health professionals and psychologists, developed the comprehensive search strategy.
- Literature screening will incorporate calibration checks to ensure a reviewer consistency rate of 90% or higher.
- A potential limitation is the limited number of studies explicitly using trauma-informed terminology to describe approaches underpinning family carer education and practical skills training.
- Studies with implicit and explicit trauma-informed frameworks will be included for synthesis and comparison.

## Introduction

 The prevalence of dementia is on an increasing trajectory globally and represents one of the most challenging 21st-century diseases.^1^ Described as a significant public health concern, dementia prevalence is estimated to reach 82 million by 2030 with around 10 million new diagnoses made every year^2^ and 153 million by 2050.^3^

Dementia is a term used to describe a broad category of over 100 types of brain disease characterised by progressive cognitive impairment and a range of neuropsychiatric symptoms underpinned by a decline in executive functioning.^4 5^ As the disease progresses the needs of the individual with dementia evolve, shifting the type and level of care unique to each individual and stage of the disease.^6 7^ For most people with dementia, care is provided informally within the family home by close friends and family; however, there is international concern surrounding growing inequalities attributed to dementia care and spiralling costs threatening to overwhelm health and social care provision.^1^

The substantial proportion of time carers spend looking after their loved one with dementia varies from 89 billion hours^8^ to 133 billion hours yearly,^1^ with carers assisting with all aspects from basic daily living tasks to complex and nuanced care. Recognised globally as an essential workforce in dementia care,^9^ carers report this role as diverse and highly rewarding;^10^ however, there are negative aspects associated with caring for someone with a progressive and terminal condition resulting in high-burden, significant risk of harm to carer health and well-being, high risk of care breakdown and burnout.^9 11^ Family carers experience global disadvantage in every domain with reduced quality of life, poorer physical and mental health^12^,^15^ and are vulnerable to strain exacerbation as they manage the day-to-day care of the person with dementia in addition to balancing responsibilities such as employment, childcare and wider relationships.^13 16^

The chronic stress and challenges carers manage as dementia progresses increases the risk of being overwhelmed emotionally and psychologically, this lowers the threshold for coping and is a significant risk for trauma.^17^,^19^ Psychological trauma is defined as ‘an event, series of events or set of circumstances that is experienced by an individual as physically or emotionally harmful or threatening and that has lasting adverse effects on the individual’s functioning and physical, social, emotional or spiritual well-being’.^20^

The journey to receiving a diagnosis of dementia, and the post-diagnosis life-altering nature of living with dementia, is now recognised within national dementia strategy publications as a trauma for the individual with the disease but also their families and carers,^21^ and this provides the gateway to re-think dementia caring via a trauma-informed lens that acknowledges the profound impact trauma exerts.^21 22^

The trauma-informed phenomenon is still in relative infancy dating back to the early 2000s and the study of adverse childhood experiences;^23^ however, the transformative impact of trauma-informed approaches to practice and care has been substantial and is now embedded within international best-practice frameworks across education and national health systems^21 24 25^ revolutionising how we understand trauma. However, although there is significant momentum surrounding trauma-informed health and social care provision, it is unclear whether the informal family caring workforce, who are providing most of the care, are benefitting from education and practical skills training that uses this best-practice approach.^26^

The plethora of interventions to support and prolong informal home caring in dementia, built on evidence surrounding carer well-being, improving resilience, quality of life, minimising stressors and so on,^27^,^29^ tend to be dominated by multi-modal psychosocial programmes usually lasting around 8 sessions over 16 weeks.^30^ Although many report encouraging immediate outcomes in carer well-being, burden and anxiety, a recent systematic review captures the wider limitation and concern surrounding the long-term impact of interventions and the sustainability of positive outcomes, particularly as the condition progresses.^31^

A scoping review has been selected because it is the most appropriate approach to identify and summarise the type and nature of evidence related to this topic. That is because the application of trauma-informed approaches linked explicitly within educational interventions and practical skills training is not mainstream within dementia. That means a scoping approach to understanding the literature will support the wider appreciation of evidence that is yet to be established.

This review will provide an overview of trauma-informed active intervention education and practical skills training that supports informal day-to-day family caring for dementia.

Preliminary searches of MEDLINE Ovid, Cumulative Index to Nursing and Allied Health Literature (CINAHL) EBSCO, the Cochrane Database of Systematic Reviews and Johanna Briggs Institute (JBI) Evidence Synthesis were conducted and no current or ongoing systematic reviews or scoping reviews on the topic were identified.

### Review questions

The two questions shown below address the review aim:

- What evidence is there of trauma-informed approaches within dementia family carer education and practical skills training within published research literature?
- What is known about the outcomes arising from trauma-informed dementia family carer education and practical skills training?

### Eligibility criteria

#### Participants

This scoping review will review studies in the context of individuals providing family home-based care for individuals with a formal diagnosis of dementia. This can be of any type and severity. Family carers of any gender, age and relationship (eg, spouse, friend) who provide unpaid care to the individual with dementia will be included.

#### Concept

The overarching concept of interest for this review is to explore trauma-informed dementia family carer education and practical skills training. All trauma-informed active intervention education and practical skills training involving planned and intentional change in skills, capabilities, attitudes, approaches and behaviour to support family carers will be considered for inclusion. This will not be limited by the mode of delivery, theoretical approach, source, frequency or length of intervention.

#### Context

In this context, the focus of the review will be on the ‘informal’ unpaid day-to-day family caring (eg, symptom management, personal care support and everyday caring akin to a progressive disease) provided by a family member or friend that takes place in the family home or residence and does not include specialist care environments (eg, nursing homes and formal care facilities).^32^ This is distinct from family caring that takes place within a care/nursing home environment. Education and practical skills training for social care support staff or within specialist home-caring contexts will not be included.

### Types of sources

Publications in English with a date range of 1990 to current, with no restriction on geographical region will be considered. This will include all types of study design, qualitative, quantitative and mixed method study design, empirical research, randomised controlled trials, systematic reviews, pedagogical papers, intervention, evaluation, pilot/feasibility studies, sociological, evidence reviews and PhD thesis. Unpublished theses accessed via The British Library Online (EThos e-thesis) and opinion papers published within peer-reviewed journals will also be included in this review. Published protocols for reviews and research will not be considered in this review.

## Methods and Analysis

The protocol has been conducted in line with Preferred Reporting Items for Systematic Reviews and Meta-Analyses extension for scoping review (PRISMA-ScR) guidance for systematic scoping reviews and registered with Open Science Framework. We used the Preferred Reporting Items for Systematic Review and Meta-Analysis Protocols checklist in the writing of our protocol.^33^ The results of the selected literature will be presented in a PRISMA-ScR^34^ flow diagram.

### Search strategy

Preliminary searches of MEDLINE Ovid and CINAHL were carried out between March and May 2023 to identify literature in this area. We will search the following databases: MEDLINE Ovid, Embase Ovid, CINAHL EBSCO, the Cochrane Database of Systematic Reviews, and Cochrane Central Register of Controlled Trials in the Cochrane Library, PsycINFO Ovid and the British Library EThOS e-theses online will be undertaken. Publications in English with a date range of 1990 to current, with no restriction on geographical region will be considered. The search outputs will be managed by Rayyan software and screened by multiple independent researchers. Results will be presented using narrative summaries and tables.

We collaborated with an experienced Academic Support Librarian to develop the MEDLINE Ovid search strategy (online supplemental appendix 1), which will be adapted for searching the other databases. In addition, we will conduct supplementary forward citation searching using Google Scholar (https://scholar.google.co.uk/) and search bibliographies of included relevant studies to identify additional relevant literature.

Reviewers have begun evaluating relevant literature, with the review expected to be completed by March 2025.

### Study/source of evidence selection

Literature returned from the database search will be exported into Rayyan software that will support the removal of duplicates, screening and selection process. Multiple independent reviewers will undertake the review. The research team will work in pairs to independently screen the literature. To ensure calibration across reviewers, a sample of 10% of papers will be reviewed by each reviewer pair to ensure 90% or above agreement. Where agreement on the relevancy of papers is below 90%, reviewer pairs will discuss discrepancies and engage in further calibration activity until 90% or above agreement is achieved.

Multiple members of the research team will independently conduct a preliminary title and abstract screen of all returned literature against the inclusion criteria and will categorise the papers as ‘Yes’, ‘No’ or ‘Maybe’. All papers categorised as ‘Yes’ and ‘Maybe’ from the preliminary search will undergo a full-text screen. If there is uncertainty or disagreement about the relevance of a paper, it will be reviewed by an independent reviewer and agreement will be made by consensus.

A preliminary search of the literature revealed that few papers explicitly state the use of a trauma-informed approach within family carer dementia education, although implicit use may be evident. Therefore, at the full-text screen, an additional step in the selection of literature will ensure research that may demonstrate the implicit use of trauma-informed approaches is not disregarded in the initial stages of review and selection. Therefore, at the full-text screen, reviewers will employ an additional criterion and only literature that demonstrates explicit or implicit use of trauma-informed approaches will be included in the final review (online supplemental appendix 3).^35^ Potentially relevant sources will be retrieved in full text, assessed in-depth and categorised as ‘Yes’ or ‘No’ with all literature categorised as ‘Maybe’ and discussed with the wider review team to resolve discrepancies.

Reviewer comments will be provided on each paper, including those excluded from the review. This process will be reported in detail within the final systematic scoping review and presented visually in PRISMA-ScR.^34^

### Data extraction

A data charting framework has been developed in line with the JBI methodology^36^ and has been piloted at the preliminary selection and screening of papers undertaken for the purposes of developing the search strategy (online supplemental appendix 2). This will be amended during the process of extraction if required and detailed in the scoping review.

Key information that will be extracted from the papers includes:

- Citation
- Author and year of publication
- Country of origin
- Aims/objectives/setting
- Methodology (participants, approach)
- Intervention (theoretical basis, modality, length of intervention)
- Trauma-informed approach (online supplemental appendix 3) (illustrative content and page number)
- Key results/outcome measure
- Reviewer comments

### Data analysis and presentation

Quantitative data extracted from the literature search will be reported by frequency counts. Graphical displays including tables and/or figures will be used to present the extracted literature and will report on types of studies, the aims, approach, length, key findings, trauma-informed approaches, and characteristics of the education and practical skills training for family carers. This analysis will also identify the ‘gaps’ in the research and with each category of analysis, the reviewer will offer a descriptive narrative to contribute towards a wider summary of the literature in this area.

Qualitative data extracted from the literature will be summarised descriptively outlining the concepts, trauma-informed nature, and characteristics of informal family caring education and practical skills training in dementia. The application of trauma-informed approaches in the context of family care outcomes will be discussed.

### Patient and public involvement

None.

### Ethics and dissemination

Ethical approval was not required for this review because the research was based solely on the synthesis of publicly available secondary data. As no primary data collection involving human participants was conducted, there were no ethical concerns related to participant consent or confidentiality. The review adhered to all relevant guidelines and ethical standards for handling secondary data. The findings from this review will be widely disseminated to ensure their impact and reach. This will include submitting the results for publication in peer-reviewed academic journals. The review findings will be shared through oral and poster presentations at both national and international conferences, allowing for broader visibility and engagement within the research community and beyond. In addition to academic dissemination, the project places a strong emphasis on engaging relevant stakeholders via seminars, and lay summary reports. Such engagement is expected to help bridge the gap between research and practice, ensuring findings are accessible and shared among relevant stakeholders.

## supplementary material

10.1136/bmjopen-2024-090202 online supplemental file 1

10.1136/bmjopen-2024-090202 online supplemental file 2

10.1136/bmjopen-2024-090202 online supplemental file 3
