# Supplementary material for: Trauma-informed family carer education and practical skills training in dementia: a systematic scoping review protocol
Source: BMJ Open. 2024 Dec 7;14(12):e090202. doi: 10.1136/bmjopen-2024-090202 (PMC11628969; doi:10.1136/bmjopen-2024-090202)
Supplement: online supplemental file 1 [file bmjopen-14-12-s001.docx]

**Appendix 1**

This search was constructed in the MEDLINE Ovid database during November 2023 and is designed to retrieve records pertaining to Trauma Informed Approaches in Family Caring within Dementia. It is constructed using a combination of Medical Subject Headings (MeSH) and uncontrolled vocabulary/keywords. It contains three core elements: terms representing dementia populations (lines 1-3), caregivers (lines 4-6), and "trauma informed" educational interventions (lines 7-14. The MEDLINE Ovid search was used the basis for all subsequent database searches included in this review.

1. dementia/ or alzheimer disease/ or dementia, vascular/ or dementia, multi-infarct/ or frontotemporal lobar degeneration/ or frontotemporal dementia/ or lewy body disease/ or mixed dementias/

2. (dementia* or Alzheim* or (lewy adj3 bod*) or (frontotemporal lobar adj3 degenerate*)).tw.

3. 1 or 2

4. caregiver burden/ or caregivers/

5. ((caregiver* or carer* or care) adj3 (sibl* or spous* or famil* or relative* or informal or unpaid or giv* or everyday or "every day" or partner*)).tw.

6. 4 or 5

7. adaptation, psychological/ or "trauma and stressor related disorders"/ or stress disorders, traumatic/ or exp psychological trauma/ or stress disorders, post-traumatic/

8. (traum* or stress* or distress* or coping or cope or resilien* or adapt*).tw.

9. 7 or 8

10. information seeking behavior/

11. health literacy/ or patient education as topic/

12. ((support* or psychological or psychoeducat* or skill*) adj3 (instruct* or intervent* or train* or strateg* or program* or dyad* or educat*)).tw.

13. ((help or knowledge or information) adj3 (seek* or needs or behav*)).tw.

14. or/10-13

15. 3 and 6 and 9 and 14
