# Supplementary material for: Trauma-informed family carer education and practical skills training in dementia: a systematic scoping review protocol
Source: BMJ Open. 2024 Dec 7;14(12):e090202. doi: 10.1136/bmjopen-2024-090202 (PMC11628969; doi:10.1136/bmjopen-2024-090202)
Supplement: online supplemental file 2 [file bmjopen-14-12-s002.docx]

### **Appendix II: Data extraction instrument**

| Category | Data to be Extracted |
| --- | --- |
| Citation | Author  Title  Year of Publication |
| Country of Origin | Location country |
| Research Summary | Aims  Objectives  Setting |
| Methodology | Participants  Approach |
| Intervention | Theoretical basis  Modality  Length of Skills Training/Education programme |
| Trauma-Informed (TI) Approach | Appendix 3 TI items 1 to 10 |
| Key results | Outcomes/Results |
| Reviewer comments | Gaps in research  Reported limitations |
