# Supplementary material for: Trauma-informed family carer education and practical skills training in dementia: a systematic scoping review protocol
Source: BMJ Open. 2024 Dec 7;14(12):e090202. doi: 10.1136/bmjopen-2024-090202 (PMC11628969; doi:10.1136/bmjopen-2024-090202)
Supplement: online supplemental file 3 [file bmjopen-14-12-s003.docx]

**Appendix 3**

**Trauma-Informed Approach**

For this review, we operationally define a trauma-informed approach as the presence of one or more of the ten criteria proposed by Sweeney and Taggart (2018). In this review, we operationally define a trauma-informed approach as meeting one or more of the ten criteria proposed by Sweeney and Taggart (2018). Originally developed for organisational change, these criteria are equally applicable to direct care settings, providing a broader, more comprehensive definition. They also aid in identifying both implicit and explicit trauma-informed principles within the interventions examined in this scoping review.

1. understands and acknowledges the link between trauma and mental health,
2. recognises social trauma and the intersectionality of multiple traumas,
3. sensitive approach to exchanges with knowledge about how to respond,
4. referral to evidence-based trauma-specific support,
5. recognises vicarious trauma and re-traumatisation,
6. prioritises trustworthiness and transparency,
7. collaborative relationships based on trust, collaboration, respect and hope,
8. strengths-based approach re-framing symptoms as coping adaptations,
9. emotional and physical safety is prioritised,
10. partnership working with trauma-survivors e.g. design, deliver and evaluate services.

The above ten-item description is a refinement for mental health contexts of the well-known SAMHSA (2014) trauma-informed principles (1. safety, 2. trustworthiness & transparency, 3. peer support, 4. collaboration & mutuality, 5. empowerment, voice & choice and 6. Cultural, historical & gender issues).
