# Supplementary material for: Trauma-informed family carer education and practical skills training in dementia: a systematic scoping review protocol
Source: BMJ Open. 2024 Dec 7;14(12):e090202. doi: 10.1136/bmjopen-2024-090202 (PMC11628969; doi:10.1136/bmjopen-2024-090202)
Supplement: Supplementary file 4 [file bmjopen-2024-090202.reviewer_comments.pdf]

PEER REVIEW HISTORY

BMJ Open publishes all reviews undertaken for accepted manuscripts. Reviewers are asked to complete a checklist review form and are provided with free text boxes to elaborate on their assessment. These free text comments are reproduced below.

ARTICLE DETAILS

Title

Trauma-informed family carer education and practical skills training in dementia: A systematic scoping review protocol.

Authors

Harkess-Murphy, Eileen, Tolson, Debbie; Cheyne, Joshua; Heron, Suzanne; Butler, Anthony; Murray, Yvonne; Mitchell, Bryan; Limond, Kath

VERSION 1 - REVIEW

**Reviewer**	**1**
**Name**	**O'Malley, Kelly**
**Affiliation**	**VA Boston Healthcare System**
**Date**	**7-Aug-2024**
**COI**	**None**

The authors are proposing to conduct a scoping literature review of trauma-informed care training/education among family caregivers, an area that has been understudied, as trauma-informed care approaches in caregiving is a relatively new area. The proposed review process is grounded in a well-established structure and the authors have thoughtfully utilized the JBI and PRISMA guidelines in this work.

One minor comment: Could the authors provide additional description of the reasons for selecting the Sweeney and Taggart (2018) operationalization of trauma-informed care? Additionally, these 10 criteria are designed for organization change (however do apply across settings and persons) and it may be helpful to provide a brief description of how trauma-informed care approaches apply at the direct care level as well.

**Reviewer**	**2**
**Name**	**Camacho , David**
**Affiliation**	**Cornell University**
**Date**	**19-Aug-2024**
**COI**	**None**

Thank you for the opportunity to review your protocol "Trauma-informed family carer education and practical skills training in dementia: A systematic scoping review protocol." Overall, this project will be a useful contribution to the literature on the use of Trauma-Informed practices with caregivers of individuals with dementia.

I have one general thought for consideration. You chose to focus on the articles that explicitly use Trauma-informed principles. Given the exploratory nature of a scoping review- I would consider including articles that implicitly use trauma informed principles for a couple of reasons:

1. If you identify both explicit and implicit use- you may then compare these types of manuscripts. This approach may increase your overall number of articles to review. You may also, find interesting differences across what gets labeled as "trauma informed" vs. not.

2. If you find that multiple articles implicitly use trauma informed principles, you may find additional support for the use of these principles.

I look forward to reading the actual scoping review!

VERSION 1 - AUTHOR RESPONSE

Source	Amendment	Status	Page No.
Reviewer 1	Could the authors provide additional description of the reasons for selecting the Sweeney and Taggart (2018) operationalization of trauma-informed care?<br><br>Additionally, these 10 criteria are designed for organization change (however do apply across settings and persons) and it may be helpful to provide a brief description of how trauma-informed care approaches apply at the direct care level as well.	For this review, we operationally define a trauma-informed approach as the presence of one or more of the ten criteria proposed by Sweeney and Taggart (2018). Although these criteria were originally developed for organisational change, they are equally applicable to direct care settings. They also help identify both implicit and explicit trauma-informed principles within the interventions under consideration in this scoping review.	Appendix 3
Reviewer 2	I have one general thought for consideration. You chose to focus on the articles that explicitly use Trauma-informed principles. Given the exploratory nature of a scoping review- I would consider including articles that implicitly use trauma informed principles for a couple of reasons:<br><br>1. If you identify both explicit and implicit use- you may then compare these types of manuscripts. This approach may increase your overall number of articles to review. You may also, find interesting differences across what gets labeled as “trauma informed” vs. not.<br><br>2. If you find that multiple articles implicitly use trauma informed principles, you may find additional support for the use of these principles.	Detailed in inclusion criteria & added to strengths and weaknesses. We will include implicit and explicit TI papers. A comparison will be made to take account of reviewer suggestions.	Pg 1
